# When you are not here, I cannot do what I want on the tablet – The use of ICT to promote social participation of young people with intellectual disabilities

**DOI:** 10.1177/17446295221087574

**Published:** 2022-04-27

**Authors:** Elisabet Björquist, Nina Tryggvason

**Affiliations:** Department of Social and Behavioral Studies, 42749 University West, Trollhattan, Sweden; Department of Social and Behavioral Studies, 42749 University West, Trollhattan, Sweden

**Keywords:** attitudes, ICT, intellectual disabilities, social participation, youths

## Abstract

Most youths use Information and Communication Technology (ICT) for socialising, but there is a discussion about whether using ICT promotes social participation for youths with intellectual disabilities (IDs). Employing the concepts of social participation and self-determination together with the youths’ perspectives, as conveyed by staff, we examine how social participation might be promoted for youths with intellectual disabilities in institutional settings in Sweden. The findings revealed three overarching themes. The first theme, *Developing skills, self-determination and becoming less reliant,* illustrates the basic use of ICT. The second theme, *Sharing events, socialising and participating with others,* draws attention to how youths take technology a step further to interact with others. The third theme, *Resources and attitudes,* concerns the youths’ need and desires for adequate support and equipment and the mindset of surrounding communities concerning ICT. Our conclusion is that ICT has the potential to promote social participation if the youths have access to personalised equipment and supportive staff.

## Introduction

Information and communication technology (ICTs) is commonly used all over the world, especially by young people. Smartphones and tablets enable many digital activities, for example, playing games, watching videos and, most importantly, communicating with peers (Stoilova et al., 2016). Even young children use digital devices and most schoolchildren in Sweden have their own smartphone (Andersson, 2020). However, the use of ICT is unevenly distributed among the population. Some youths are left behind, due to lack of Internet access, devices and/or the ability to handle the technology. Hence, disability researchers argue there is an emerging digital divide, in which some individuals and certain groups are at risk of being marginalised in a digital world. Youths with intellectual disabilities are one example (Alfredsson Ågren et al., 2020; Borgstöm et al., 2019; Chadwick et al., 2013; Ok, 2018). This divide can depend on different socio-economic conditions but also the fact that devices and technologies are mostly developed for non-disabled youths. Furthermore, there are shortcomings both in organisations and among professionals in terms of education and attitudes towards technology (Chadwick et al., 2013; Näslund & Gardelli, 2013; Ramsten, 2018). According to Article 9 of the UN Convention on the Rights of Persons with Disabilities (UN, 2006), persons with disabilities have the right to access ICT, including the Internet (Article 9g). Furthermore, in Sweden, youths with intellectual disabilities in need of support are entitled to support by the Act concerning Support and Service for Persons with Certain Functional Impairments (LSS). This law is based on the principle that every individual has the right to participation, self-determination and influence (SFS 1993: 387). Youths with intellectual disabilities are a heterogeneous group, but many need help managing situations such as Internet activities (Kennedy, 2010). Depending on individual challenges, the kind of support needed can therefore differ.

According to previous studies, tablet devices, smartphones and/or individually adapted applications (apps) are a quotidian necessity for many youths with disabilities. Assistive technology especially designed for people with disabilities has been used for many years and can promote independence and participation. Technology for Alternative Augmentative Communication (AAC) is used for people to support understanding and speech, and today there is a plethora of AAC-tools, some of them also available as apps (see e.g. Murphy & Cameron 2008; Stans et al., 2019; Stewart et al., 2018). In this area, development is progressing rapidly and new digital tools are being tested, such as apps that support communication, thus facilitating participation for young people with complex communication difficulties (Babb et al., 2021; Caron, 2018). Such assistive technology can also facilitate everyday activities and can lead to less reliance on parents or professionals (Buchholz et al., 2018; Stephenson & Limbrick, 2015). In addition, Söderström et al. (2019) found that assistive technology affects the lives of young adults with intellectual disabilities by increasing predictability and facilitating self-determination in everyday activities, but can also be perceived by users as stigmatising. People in general use digital tools found on ordinary tablets and smartphones to facilitate everyday life. There are apps for remembering functions and shopping lists, for example. By using the same digital tools as others, young people with disabilities might experience less stigma than they do when using assistive technologies (Heitplatz et al., 2021). Still, this requires access to the technology and support in using it if and when the individual needs and wants it (Alfredsson Ågren et al., 2018, Darcy et al., 2016).

Whether the use of ICT promotes social participation for youths with intellectual disabilities or not is debated. On the one hand, research shows that using ICT enhances self-confidence, self-determination and makes youths less reliant on support staff, which in turn might lead to enhanced participation. Indeed, Barlott et al. (2019), who interviewed people with intellectual disabilities, found that using ICT created possibilities in important areas like maintaining contact with relatives and friends and developing new relations. Using ICT also enabled a cultivation of interests and the organisation of everyday life (Alfredsson Ågren et al., 2018, Darcy et al., 2016).

In contrast, there are researchers questioning whether ICT per se contributes to social participation. Seale (2014) questioned the Internet’s empowering effect on youths with intellectual disabilities, noting a difference in how staff in their environment think about the risks and benefits of using ICT. Whether this affects youths’ opportunities to develop self-determination was found to depend on attitudes towards risk-taking among staff. Molin et al. (2017) interviewed youths with intellectual disabilities in Sweden to explore whether online activities had an emancipative impact on youths’ identity development, which might lead to enhanced social participation. They found that only in a few cases did online activities contribute to positive identity development. Instead, youths’ Internet activities could contribute to both reduced and increased participation, depending on how well youths could manage risks. Staff may have an over-protective attitude, afraid that youths with intellectual disabilities could be exposed to cyberbullying or other cruelties that are more common among youths with disabilities than among their non-disabled peers (Borgström et al., 2019; Swedish Media Council [Statens medieråd], 2017).

This paper focuses on youths in LSS settings for people with disabilities. According to the LSS law, individuals with, for example, intellectual disabilities and/or with the autism spectrum disorders, the right to support in their daily lives (SFS 1993:387). LSS support can be provided individually or by activities in social care settings. According to the Swedish National Board of Health and Welfare (2019), 95% of children receiving LSS support have an intellectual disability. Many individuals with intellectual disabilities need to be given the opportunity for alternative ways to communicate, both due to limited abilities to communicate verbally, but also to support understanding (see e.g. Murphy & Cameron 2008; Stans et al., 2019; Stewart et al., 2018).

Previous research on the use of digital technology for youths with intellectual disabilities in institutional environments has primarily concerned the perspectives of support staff (e.g. Isaksson & Björquist, 2020; Ramsten & Blomberg, 2019; Ramsten et al., 2019). Furthermore, digital technology in relation to youths with intellectual disabilities has mainly been discussed in the context of social media (e.g. Caton & Chapman, 2016; Molin et al., 2015). At a time when digital technology is becoming increasingly important, there is a need for more knowledge concerning how youths with intellectual disabilities in social care settings can make use of this technology. The aim of this paper is therefore to examine how the use of ICT in LSS settings might promote social participation for youths with disabilities.

### Social participation, self-determination and attitudes

The concept of participation for people with disabilities has been discussed by numerous researchers and can be defined in several ways depending on the context. For youths with disabilities, WHO’s (2007) International Classification of Functioning, Disability and Health for Children and Youth (ICF-CY) defines participation as a person’s involvement in life situations. This involves what a person *does* together with others: how *involved* they are and what they feel is *meaningful* (WHO, 2007; Chang & Coster, 2014). This can mean having access to what other youths have and thus becoming more involved in everyday activities. However, in this article about youths with intellectual disabilities in an institutional context, the concept ‘social participation’ will be used. Based on a content analysis of several articles concerning social participation, Levasseur et al. (2010 p 2148) propose six levels of participation. 1) preparation for participation by doing an activity 2) being with others 3) interacting with others 4) doing an activity with others 5) helping others and lastly 6) contributing to society. Social participation is defined ‘as a person’s involvement in activities that provide interaction with others in society or the community’. This means that social participation must specifically include involvement with others. According to Levasseur et al. (2010), this is what distinguishes participation (Levels 1–2) from social participation (Levels 3–6).

Closely linked to social participation is self-determination, which can be understood as being an actor in one's own life. Several studies have shown that self-determination is important for youths with disabilities to achieve well-being and quality of life (see e.g., Liang et al., 2020; Wehmeyer, 2020). For youths with intellectual disabilities who need support from staff, it must not imply complete independence but rather becoming less reliant on staff support in certain situations. Having the opportunity to develop social participation and self-determination during childhood and adolescence is therefore important for quality of life both here and now and later in life.

Prerequisites for both social participation and self-determination in youths needing support are the staff's attitude and organisational resources for providing that support. Central to young persons with intellectual disabilities are supportive adults with positive attitudes and the possibility to have a say, negotiate and, according to individual ability, be involved in decisions (Chadwick et al., 2013; Ramsten, 2018; Seale, 2014). A positive, permissive attitude towards these youths’ use of digital technology within care settings is of great importance. Staff members’ attitudes regarding whether youths should be exposed to risks may have a significant impact. Risk-taking is often interpreted as something negative, but positive risk-taking entails a potential for personal growth and development. The attitudes espoused by their environment are therefore decisive for youths if they are to gain greater control over their lives, which can lead to increased self-determination and social participation (Chadwick et al., 2013; Ramsten, 2018; Seale, 2014).

## Methods

The empirical data in this article were collected during a collaborative project between the university and six Swedish municipalities. The project included interviews with staff members and youths spending time in the settings in accordance with the LSS law. The communities’ part of the project aimed at expanding the possibilities for using ICT in disability care settings involving the acquisition of ICT equipment like tablets as well as ICT training for staff members. The care settings in this case are ‘short-stay homes’ and ‘daily activity centres’. The purpose of a short-stay home is to offer leisure activities and a change of environment for youths, or respite for their ordinary caregivers. Short-stay homes can offer opportunities to spend time with peers, play and have new experiences. Daily activity centres aim to offer an alternative to ordinary workplaces and simple tasks like handicrafts and gardening but also occupational and physical therapy. During a day in these settings, youths also have individual free time when they can choose an activity (SFS, 1993; Sjöberg, 2016). Daily activity centres target people who have finished school, while short-stay homes are for both children and adults; thus, this paper focuses on children and youths up to age 30.

To provide insight into the youths’ perspectives, focus group interviews were conducted with staff who in turn had spoken to the youths. The staff`s experiences and perceptions are thus derived from conversations that they had with the youths. To gather information on the perceived views of the youths irrespective of communication abilities, it was considered best that staff who knew them and their way of communicating were valuable sources of information. This is inspired by a method previously used to capture vulnerable children’s views on social services (Rosenberg et al., 2012). It was also seen as important that conversations took place in a familiar context for both staff and youths, where ICT was used. In preparation, a conversation guide was created by the researchers and LSS organisation representatives, ensuring that similar themes were covered: *the access to and the type of technology, apps and programs used*, *situations and reasons for using ICT* ; *how the youths interacted with others by using ICT, and what kind of support they required*. All conversations with the youths took place on a voluntary basis within the context of ordinary activities in the settings. Five staff members from short-stay homes and three from daily activity centres talked to one young person each on one or more occasions. All of the staff members except one were females and five had special assignments within the project mentioned above, to supervise LSS staff in the use of computer tablets. The youngest of the youths with whom the staff members had conversations were 10 years of age, two were teenagers, four were around 20 and the oldest 30 years of age.

The purpose of a focus group interview is that participants through interaction are stimulated to immerse themselves in a topic (Krueger & Casey, 2009). In this case, it was based on the staff’s interpretation of what they learnt from their conversations with the youths. During the focus group interviews, the participants were asked to describe what the youths had told them concerning the above themes. The participants had notes from their conversations and in some cases this also included quotations. At the end of the interview, themes that had emerged was summarised and discussed together with the participants. Two focus group interviews with different participants, lasting 60–80 minutes, took place. With consent, the focus group interviews were audio recorded and transcribed verbatim by the authors. It is important to point out that the empirical data in this paper is based on the focus group interviews and not on direct statements from the youths themselves.

### Data analysis

The data were analysed using thematic analysis. This is a method for identifying, analysing and reporting patterns (themes) in data (Braun and Clarke, 2006). Inductive analysis was performed in line with Braun and Clarke (2006), following six steps. To become familiar with the material, both authors first read and reread the transcribed interviews, noting ideas and reflections. In the next step, interesting passages in the text were selected, generating initial codes. For example, all quotations about how ICT could facilitate communication were collated. In the third step, all codes were interpreted, keeping in mind the question of whether ICT could promote social participation. Thereafter, all relevant codes were sorted into potential themes and then organised into sub-themes. The fourth step involved reviewing and refining the potential themes and the fifth step involved defining and naming themes and sub-themes resulting in three overarching themes, each of which had two sub-themes. In the last step, we created texts describing the interpreted meaning of the quotations, which were finally discussed in relation to previous research and the theoretical perspectives of social participation. Steps 2–4 are visualised in Table 1.

**Table 1.** Example of the analyse process.

Interesting passage	Code	Sub-theme	Theme
…she who I talked to described how difficult it can be to send an SMS…it can so easily go wrong, how to get the letters in order – and yet you can get help from autocorrect, get support in finding small words…(FG1)	Facilitating communication	Spontaneous communication using ordinary devices	Developing self-determination and becoming less reliant

## Results

The findings in the study are organised into three overarching themes. The first theme, *Developing self-determination and becoming less reliant*, illustrates the basic and more individual use of ICT. The second theme, *Sharing events, socialising and participating with others*, draws attention to how the youths take their use of technology a step further to interact and broaden their views. The third theme, *Resources and attitudes*, concerns the youths’ requests for adequate equipment and support and the mindset of the surrounding community concerning use of ICT for youths with intellectual disabilities.

The result is presented with quotations referred to as Focus Group (FG) 1 or 2 and not by referring to individual staff members participating in the focus groups.

### Developing skills, self-determination and becoming less reliant

The following theme illustrates that the use of tablets and ICT opens for spontaneous activities, the ability to communicate, expressing oneself and developing one’s own interests and being able to carry out activities with less reliance on the staff.

### Spontaneous communication using ordinary devices

Being able to communicate, to successfully convey thoughts through words is an important part of social participation. Some of the focus group narratives concern youths who can communicate orally but still need tools to strengthen verbal skills. Pictures and apps on the tablet enable spontaneous communication. One of the staff members said that a boy with limited verbal ability had told them that he uses the camera on the tablet to take pictures. These pictures help him mediate what he has done in different places during the day.

Communicating does not only concern speech but also writing. Another staff member explained that a girl uses a regular smartphone with features available to anyone, instead of using assistive technology. She explained that the functions on the smartphone can be helpful with spelling when writing text messages:

*… she who I talked to described how difficult it can be to send an SMS …it can so easily go wrong, how to get the letters in order – and yet you can get help from autocorrect, get support in finding small words… (FG 1)*

Although an app is not an obvious assistive tool, it can still be associated with assistive technology. One boy told the staff member that he is interested in doing things on his phone, like everyone else does, but is very negative to all forms of assistive technology:

*It’s just games he’s interested in. In school, he has other programmes for learning things, and he thinks that's crap, he says. So [learning] is not ‘the thing’ [with the tablet or smart phone], it's just for playing his games. (FG 1)*

These examples illuminate situations when the youths, by using ICT, can express themselves and communicate more spontaneously, with less support from others. The youths whom the staff had talked to seem to prefer using ordinary devices and are still negative to technology specially adapted for people with disabilities.

### Becoming less reliant on staff

According to staff members, ICT devices offer opportunities to do things, when the youths want to do something, without having to wait for help from staff. Doing things on their own also makes it possible to focus on skills, rather than shortcomings. Several examples illustrate how the youths experience ordinary digital tools as helping them to become less reliant on staff. One example concerns a boy who expresses satisfaction with getting instant help from the Internet. A similar story retold by another staff member concerns a girl who enjoys the immediate possibilities that the Internet offers. Instead of having to wait for staff support or a scheduled activity, she told the staff member that she can do whatever and whenever she wants:

To dive deep into an interest without being dependent on – ‘now we should go to the library together and borrow books’ … If it is music, ‘now I want to show how to dance, then you can get into it’. (FG 2)

Surfing the Internet not only enables spontaneous searching for information and cultivating areas of interest but also contributes to a higher degree of self-determination. Another dimension of self-determination is carrying out activities with support from ICT, instead of staff. Being able to manage activities, at least partially, without support from staff seems to be motivating and gives the youths a sense of self-control. One example mentioned by a staff member had to do with a boy doing some cooking at the short-stay home:

*He says it is fun to be able to cook and that [the tablet] gives instructions instead of the staff saying everything: ‘Now you take out this or that’…it is the iPad that says it instead of the staff. (FG 2)*

Even if the youths are apparently happy to manage on their own, it is worth mentioning that staff are nearby for support, but ICT seems to allow them to act in the background. The above examples highlight the importance of mastering ICT to become more self-determining and thus less reliant on staff.

### Sharing events, socialising and participating with others

Comprehending and handling technology seems to make youths feel more self-confident, which in turn can result in social contacts. The following theme illuminates how ICT can support interactions both inside and outside the settings.

### Teaching others, sharing events and preparing for future participation

Being able to both understand and get a grip on different kinds of ICT, and then also using this to teach others and socialise, seems to increase both self-confidence and self-esteem.

One staff member told us about a boy who emphasises that his disability can be an asset when it comes to ICT; he thinks it makes him more skilled at using digital devices than his peers. This seems to make him proud and keen to share his knowledge and socialise with others:

*He said he was very happy that he had Asperger's, he returned to this several times, that it was this that made him so good. That it is enough that he looks at an app once: ‘Then I just remember’, he said. And he was happy to teach the other children. (FG 2)*

Others use the technology in a creative way to develop innovations that can be used together with other youths in the setting. A staff member provided one example of this concerning a girl working in a gardening group at a daily activity centre, her task being to instruct the other youths in the group in cultivation. First, she interviews each group member about their specific task. Then, with support from a programme on the tablet, she develops a theme map with instructions that anyone in the group can use in their gardening work.

These examples show how ICT can support interaction between peers when they help each other. Another example that illuminates how ICT enhances social interaction is when a boy who is very interested in online gaming got the idea to create an offline board game based on the same characters. He uses the computer to produce a game and invite others to play. *Developing games is also something he wants to work with as an adult*. Another conversation with a girl working at a daily activity centre, as narrated by a staff member, also illustrates how knowledge of digital devices relates to thoughts of the future. The girl, who defines herself as an assistant nurse, wants to develop her skills in sign language and find a way to do this using the tablet:

*…she wants to know a lot of sign language because she thinks she will work with it in future […] So she practices language by looking at [the tablet] […] she searched for words she did not know, saw how the person signed the word and then she mimics it and then she uses it. (FG 1)*

The examples above show how the youths link their interests and willingness to learn about ICT to plans for a future working life. Both these examples show how the youths prepare for participaton in society. For others, this is not as pronounced or even mentioned. Still, it is obvious that using the devices and online contacts provides a chance to deepen one’s knowledge in areas of interest. Several participants in the focus groups had experienced how ICT helps youths dig deeper and shares their interests by improving their capacity to communicate in English. One girl has quite a narrow interest in a specific kind of animal. The girl spends a lot of time doing online research and interacting with others who share her interest. She has even had correspondence with an American expert in the field.

There are several other examples in the focus group interviews showing that youths develop their ability to communicate in English thanks to ICT. Two of the other youths mentioned, one of whom comes from an English-speaking country, share an interest in gaming and English is their common language while participating in the gaming world:

*…when they talk about games they use English, it's mostly when they watch short video clips or whatever it is they do – that's when they speak English, when they have to explain their games. It's something they'll do in the future; they'll work together then. He really feels that he is very good at this. (FG 1)*

The stories above show that by mastering the technology the youths learn new things, boosting their self-confidence, which opens doors to interaction and social participation.

### Online participation

To what degree and in what way the youths are involved in online activities outside the setting is something the staff participating in the focus groups have limited knowledge of, but some online activities are used at the settings. Skype is used by some of the youths, mainly for keeping in touch with their families. Skype is also preferable to text messaging for those who have trouble spelling. For one girl whose family is abroad, this is a good way to connect with them, especially since she can manage it on her own. Others told the staff they used social media outside the setting. One staff member told us about a girl who said that she keeps in touch with friends only through social media because she can’t find time or energy to meet them in real life:

*,,, it is mostly Messenger and Facebook… it is an opportunity to keep social relationships alive. As she says, ‘You may have worked a whole day and then you come home and do the laundry and stuff. Then you might be tired, so then, you kind of do not have the strength’. (FG 1)*

These stories reveal that youths in disability care settings, in the same way as other youths, call, text, skype, surf, watch YouTube clips and play games, but to a limited extent. Partly this is due to a lack of devices; the few they have are to be shared by everyone.

The staff also provided examples of youths who have opted out of online activity because it’s too difficult if they are not able to spell or maintain the speed required in games. For one girl, these limitations meant that she no longer plays with others:

*She played some football games and stuff. I asked her if she was playing with others and she said that she has tried, but that it goes so fast. Then it’s the thing with the spelling and then she obviously doesn’t follow, so then she has opted out of that part. (FG 1)*

To be able to connect to others online, many of the youths are dependent on staff support. Even though technology is helpful in many ways – like having autocorrect when sending texts – the youths still need a helping hand to get it right. How staff respond to youths’ need for support when using ICT and what resources are offered will be discussed in the next section.

### Resources and attitudes

This theme concerns how the youths describe their opportunities as well as obstacles to getting access to technical equipment and how resources and attitudes influence their possibilities to use it.

### Technical and organisational barriers to participation

A basic prerequisite for youths in disability care settings who want to learn and enjoy the benefits of ICT is that technology equipment exists, and that it actually works. The stories the youths told, mediated by the staff, show that this is not always the case. The youths express dissatisfaction on several accounts concerning how the technology fails:

*When the Internet doesn't work: ‘Ahhh, we have no Internet!’ Or when the mouse is broken … There's a lot about when something breaks. For example, when we are downloading an app, it goes too slowly… It may be that the Internet is down because the router is broken or because the sim card didn't have a code. (FG 1)*

Most digital functions require being online, but some settings lack network connections and in others it works poorly, as the quote above illustrates. In addition, online opportunities vary between the youths depending on whether they have access to external accounts or not. Many of the youths have no devices of their own at home, which means they are dependent on the equipment at the settings to complete their projects. If the technical obstacles are numerous or too hard to surmount, this could mean lost interest:

*Many do not have tablets at home and want to print! Now we print their digital books, so they get them in paper form to take home. But for a long time we have not had the opportunity to do that, and it has been functional mailboxes and such, and the girl I talked to thought this was no good. She told me she lost a bit of her enthusiasm. (FG 1)*

The lack of devices means that youths must share with others. At several of the settings, this is solved by scheduling the use of devices. In those cases, each youth only has a limited and specified time when they have access to the device. Furthermore, this means that the equipment is not personalised and thus more difficult to handle. Having their own equipment is therefore on top of many youths’ wishlists:

*… this girl has no iPad of her own, she explicitly says that she wants to buy her own. And at work, she says that since everyone is entitled to use it [the iPad at the setting], she thinks it's frustrating that it’s according to a schedule. She said: ‘I want a new computer since it is five years old and from school, I want a tablet, I have never had one, and I want to update my phone … (FG 1)*

All in all, the focus group interviews with the staff showed the importance of having technology that really worked – from the youths’ perspective. Deficiencies in technology had a direct effect on the youths’ opportunities and desire to develop their digital abilities and participate in the digital community.

### The importance of positive and permissive attitudes

Young people who need support are dependent on staff wanting to help. The following sub-theme highlights not only what the youths conveyed but also how the staff perceive the situation.

The youths requested just-in-time support. A prerequisite for this was the staff’s knowledgeability in ICT and their positive attitude. From the focus group discussions, one can see that there are differences in attitudes and how permissive the staff should be when it comes to the use of ICT and how interested and knowledgeble the staff are:

*The knowledge can be lacking when the staff who is going to work that day do not know how to use the technology…Yes, you have heard that many times ‘when will hmm [the right staff] come so we can do this’, you know… (FG1)*

Several participants in the focus groups agree with this statement and one participant shared what a girl had said:

*She says, ‘When you're not there, when your kids have been sick so often… When you're not there, I can't do it’. Then she gets discouraged and loses her enthusiasm. (FG 1)*

The attitude towards ICT seems to differ both between and within different settings. This was explicitly revealed during the discussion in the focus groups, but also through their recounting of what the youths had conveyed. On the one hand, there was a permissive attitude towards the youths’ use of tablets. It was compared to being physically active or outdoors in the fresh air:

*This guy really fancies doing things, being outdoors and walking… He usually takes it [the iPad] out immediately when we come back to the short-term home, but we have not limited him because we feel that we are still out doing activities, not that it takes away from other stuff to sit with the iPad or anything… (FG 1)*

The quote above shows a discussion about the use of iPads vs other activities that may be considered preferable. On the other hand, the staff member telling the story seemed to argue that if the boy she interviewed did all the other things he needed to do, it was okay to spend time on the tablet. Although the participants in the focus groups had a positive attitude towards using ICT, the quotation above reflects a more restrictive attitude.

## Discussion

The purpose of the study was to investigate whether using ICT in disability care settings could promote social participation for youth with intellectual disabilities. From a broader perspective, this is not only about promoting social participation in the settings but also in other life situations. In the following section, the results of the study will be discussed in relation to the taxonomy of social participation as proposed by Levasseur et al. (2010 p 2148). Using this taxonomy, we want to shed light on how youths’ social participation might increase by using ICT.

The first theme that emerged from the results concerned how developing skills in ICT could contribute to enhanced self-determination and less reliance on staff. The availability of ICT enabled youths to master more ways of expressing themselves spontaneously and creatively. What was previously an analogue activity to support communication, could now be done by using a digital technique. This is supported by previous research showing that the use of tablets for example, in many situations can facilitate spontaneous communication for youths with disabilities (Buchholz et al., 2018; Heitplatz et al., 2021; Isaksson and Björquist, 2020; McNaughton and Light, 2013). Another aspect that also emerged in our results was that the youths, in line with previous research, preferred technology used by youths in general. For example, Heitplatz et al. (2021), who interviewed adults with intellectual disabilities living in residential settings, found that they also had the feeling of being like everyone else when using ordinary smartphones. The importance of being like others is understandable considering previous research that has shown that ICT used by people in general, in contrast to assistive technology, can be perceived as less stigmatising for many youths with intellectual disabilities (Söderström & Ytterhus, 2010). The young people in our study also belong to a generation that grew up with digital technology as an obvious part of everyday life (Andersson, 2020; Stoilova et al., 2016), which makes it even more plausible that using assistive technology can be perceived as stigmatazing.

Overall, using ICT, when available, seemed to enhance self-determination and made it possible to focus on abilities rather than shortcomings. Another important aspect was that with the support of ICT, the youths became less dependent on staff to cope with everyday activities. This is also in line with previous research (e.g., Burckley et al., 2015; Darcy et al., 2016; Isaksson & Björquist, 2020; Ramsten et al., 2019). The examples mentioned can be interpreted as the first step towards participation by providing the opportunities and skills needed for more extensive participation. According to Levasseur et al. (2010), the first and second levels of participation include *preparation for participation* and *being with others,* the latter being one of the ideas behind organised activities in settings for youths with intellectual disabilities (SFS, 1993). Findings in this paper reveal that using ICT makes it possible for the youths to develop skills beyond these basic levels. Levasseur et al. (2010) argue that the difference between participation and social participation is that the latter must include interaction with others when doing activities in a community. ICT makes it easier for youths to communicate with others by using pictures, spoken language and foreign languages, and this enhances the next level of participation: the ability to socialise and interact with others (Levasseur et al., 2010).

The second theme is even more convincing in showing how ICT can support youths’ interaction. Several studies show that online activities, like using social media, provide opportunities for social interaction (e.g. Borgström et al., 2019; Heitplatz et al., 2021). However, our results also show how not only online but also offline activities with support of ICT can facilitate interaction. For example, the youths’ skills in ICT can be used to help others, like when the girl with the help of the computer creates materials that can be used in activities for other youths. In addition, this creates social interactions, strengthens capacities and boosts self-confidence. This seems to lead to ideas about future jobs which in the long run might result in participation in society. Further, our results show that online activities create opportunities to get in touch and maintain contact with others both within and outside the settings. Isaksson and Björquist (2020), who examined how ICT was used in institutional settings for youth with intellectual disabilities also found that digital tools enabled interaction both within and outside the settings. Our results provide examples of the fourth level of participation *doing an activity with others* and also the next step *helping others* (Levasseur et al., 2010). The fifth level, helping others, implies performing activities where collaboration is needed to reach common goals. Overall, these examples show both the fourth and fifth levels, which is an interesting finding in that ICT not only seems to support youths’ participation on a basic level but also strengthens social participation on a higher level.

The final theme concerns the issue of access to ICT resources and staff attitudes towards youths' use of the technology. From the youths' perspective, the equipment available in the settings is insufficient, which is unfortunately consistent with previous research (Alfredsson Ågren et al., 2018, Darcy et al., 2016; Näslund & Gardelli, 2013). In several places, there is no access to the Internet and the available devices are too few and must be shared with others. Access to equipment and lack of networks are according to The Swedish Authority for Participation [Myndigheten för delaktighet] (2020) common in many settings for people with intellectual disabilities. Further, access to personal digital equipment is also a question of having economic resources, and youths with intellectual disabilities do not always have the financial means to buy their own (ibid). An important finding is therefore the need for personalised devices to make it possible to utilise the potential in ICT. An equally important aspect is the youths' need for support from staff who are both knowledgeable and have a positive attitude towards supporting the youths when they need it the most. However, earlier studies show that there is a complexity considering the support staff’s ambiguity towards whether young people with intellectual disabilities should be supported in their use of ICT for social interaction or if they should be protected from risks associated with ICT use (Ramsten et al., 2019; Ramsten & Blomberg, 2019). For example, Ramsten et al. (2019) put forward that the staff's implicit moral values can have an impact on what opportunities for participation young adults with intellectual disabilities can have in everyday life and what kind of support they are offered.

This shows that using ICT does not create social participation per se; rather, this depends on whether the environment offers appropriate conditions for providing support. Evidently this requires not only enough devices and support staff knowledge of ICT, it also above all requires a positive attitude and willingness of the staff to provide support just-in-time. Based on our results we can assume that it is through supportive staff, with positive and permissive attitudes, that some of the youths have been able to reach a higher level of social participation by using ICT (Levasseur et al., 2010).

What is required for increased social participation for youths with intellectual disabilities? Previous research shows that people with disabilities generally have poorer access to the Internet at home than the rest of the population (Scholz et al., 2017), which makes it extra important to have it in their other environments, such as social care settings. According to the youths, several of the disability care settings lack a stable Internet connection, access to adequate and personalised devices that enable spontaneous access and available supportive staff. These are basic conditions determining whether ICT can be used at all, especially for social participation. This is remarkable considering all the settings in the study have been part of a project where ICT equipment and educated staff have been available. Even if these settings have good resources, when it comes to devices and knowledgeable staff, this is clearly not enough in the eyes of the youths. As per the title of this paper, youths are dependent on ‘the right staff’: those who have a positive and permissive attitude towards the youths’ use of ICT. The importance of staff members’ attitude towards ICT is supported by earlier research (e.g. Borgström et al., 2019; Seale, 2014). We therefore believe that there is a risk that if the young people are referred to staff who don’t have the right attitudes, it can counteract participation. This is also something Ramsten and Blomberg (2019) highlight as a hindering factor for young people’s rights to full participation in society when it comes to the use of ICT. Furthermore, Ramsten et al (2019) argue that implicit moral values of staff regarding the use of ICT, especially when it comes to social media, can prevent social participation for young adults with intellectual disabilities. Being able to use the Internet is for many young people with intellectual disabilities a way to be involved in society. However, although social media did not turn out to be the primary focus in the results of our study, having the opportunity to communicate regardless of abilities is an important basis for participation. This is a prerequisite regardless of whether it applies to activities within disability care settings or with others in outside world through social media. As previously mentioned, development of ICT tools adapted for people with disabilities is progressing rapidly. Current studies show examples of how apps for communication can increase social participation for young people with intellectual disabilities (e.g. Babb et al., 2021; Caron, 2018) and giving supporting staff access to knowledge about new innovations is therefore essential.

## Conclusions and implications for practice

The specific aim of this article has been to examine how use of ICT in LSS settings might promote social participation for youths with disabilities. One overarching conclusion is that ICT has the potential to promote social participation, although it is not the ICT per se that will make this happen. Evidently youths need digital technology equipment to be able to make use of ICT. There must also be an Internet connection in settings for people with disabilities since all digital activity requires it. However, the youths referred to here have underscored the importance of having their own devices. Most people find it easier to use their own digital equipment. This is especially true for youths with intellectual disabilities, who more easily can handle personalised smartphones, in contrast to shared devices. With the rapid technical development, it can also mean the opportunity to have adapted apps for individual needs in the young person’s own equipment. Most importantly, their own equipment can be used wherever they are, in care settings, at home or elsewhere – and thereby make them like young people in general. At the same time, this issue concerns youths who need support in everyday life. This is why staff not only need to have knowledge of ICT but also have both a positive attitude towards it and readiness to provide tangible support – right when this is required. If these conditions are present, ICT can make it possible for youths with intellectual disabilities to develop self-determination and at least in some situations become less reliant on staff. This can lead to increased social participation for youth with intellectual disabilities.

## Limitations and future research

The empirical data of the study originates from conversations that staff have had with the youths and were conducted by staff and not directly by researchers, which might be a limitation. There are always two sides of the coin. Through this method, youths could have their voices heard, regardless of their communication abilities. However, this could mean that the youths were disingenuous with staff they are dependent on. Therefore, we suggest future studies to collect empirical data directly from youths themselves. To be able to interview youths who need Alternative and Augmentative Communication (AAC), researchers need to develop their knowledge in AAC methods. This might be done by using ICT.
